# Effective Stiffness of Thin-Walled Beams with Local Imperfections

**DOI:** 10.3390/ma15217665

**Published:** 2022-10-31

**Authors:** Natalia Staszak, Tomasz Gajewski, Tomasz Garbowski

**Affiliations:** 1 Doctoral School, Department of Biosystems Engineering, Poznan University of Life Sciences, Wojska Polskiego 28, 60-637 Poznań, Poland; 2 Institute of Structural Analysis, Poznan University of Technology, Piotrowo 5, 60-965 Poznań, Poland; 3 Department of Biosystems Engineering, Poznan University of Life Sciences, Wojska Polskiego 50, 60-627 Poznań, Poland

**Keywords:** numerical homogenization, local imperfections, thin-walled beams, finite element analysis

## Abstract

Thin-walled beams are increasingly used in light engineering structures. They are economical, easy to manufacture and to install, and their load capacity-to-weight ratio is very favorable. However, their walls are prone to local buckling, which leads to a reduction of compressive, as well as flexural and torsional, stiffness. Such imperfections can be included in such components in various ways, e.g., by reducing the cross-sectional area. This article presents a method based on the numerical homogenization of a thin-walled beam model that includes geometric imperfections. The homogenization procedure uses a numerical 3D model of a selected piece of a thin-walled beam section, the so-called representative volume element (RVE). Although the model is based on the finite element method (FEM), no formal analysis is performed. The FE model is only used to build the full stiffness matrix of the model with geometric imperfections. The stiffness matrix is then condensed to the outer nodes of the RVE, and the effective stiffness of the cross-section is calculated by using the principle of the elastic equilibrium of the strain energy. It is clear from the conducted analyses that the introduced imperfections cause the decreases in the calculated stiffnesses in comparison to the model without imperfections.

## 1. Introduction

Thin-walled cross-sections have been used in structural engineering for decades [1,2,3]. The smallest thickness in a typical thin-walled member reaches 1.5 mm. The thin-walled cross-sections are produced from sheet steel, usually with higher strength, therefore steel such as S320 or S355 is often used to form particular thin-walled beams. The sheets are cold-bended to obtain the desired cross-section; therefore, due to production methods, there are obvious limitations on the shape of thin-walled cross-sections. The most popular in structural engineering are Z or C profiles [4,5]; however, Sigma profiles [6,7] have also recently been used. Structural members with thin-walled cross-sections are used in light structures as one of the first load-bearing elements; for instance in roof purlins, but also as secondary load-bearing elements in steel sheds, or supporting structures in photovoltaic installations, such as rafters or columns.

Recently, due to the increasing popularity of energy from renewable sources, but also due tothe geopolitical situation in Europe, solar-power photovoltaics have gained more interest. Of three main sources, wind, hydro, and solar power, the solar source is the most rapidly developing. In 2008, electricity generated from solar power was 1% in total, while in 2020, the value was 14% [8]. Since large solar farms are usually located on the ground, they need a relatively economical and reliable system of fastening to the foundation, which is most often made of thin-walled structures.

The main advantage of thin-walled sections is that their application allows for the optimal use of the construction material [9]. The relation of the load-bearing strength to weight of this construction element is high, compared to other typical steel structures from hot-rolled elements, such as I-beams, rectangular, or square pipes. Furthermore, nowadays, when the price of steel is breaking new records in Europe, the use of thin-walled steel structures is a reasonable and efficient way to avoid significant overinvestment due to the rapidly increasing price of the material. Furthermore, due to low weight, the mounting of the structure is faster and easier—heavy construction and transport equipment is not required.

Apart from all of the above-mentioned advantages, the biggest disadvantage of the thin-walled cross-sections is their vulnerability to imperfections, both material and geometric. The imperfection may lead to local [10] or global [10,11,12] buckling even for relatively small loads. The buckling results in the lower stiffness of the structure due to compression, bending, and/or shearing forces.

If a structure design is very complex, a three-dimensional finite element method (FEM) model is required to take into consideration the full integrity of the structure and the direct transmission of the loads onto lower substructures. Using FEM and creating a full 3D model for very complex structures would not only be laborious but also very time-consuming in terms of modeling time and computational cost [13]. In such cases, the complex structures are often modeled with a mesoscale approach, in which some parts of the structure are simplified to reduce the cost of the computations without losing the required accuracy [14,15]. Simplification may be obtained through adopting the numerical homogenization technique, which will be able to represent the behavior of the substructure with sufficient accuracy. In such cases, the application of numerical homogenization techniques may significantly speed up the computations. The reduction in the size of the FEM equation system is usually several times [16].

In the literature, there are various homogenization methods. One approach uses the equivalent of the deformation energy [9,17,18,19,20,21]. The authors of this paper wrote several papers regarding the numerical homogenization technique for plates, which were based on a concept from Biancolini [17]. This method was extended and successfully used for the homogenization of layered sections of shells in corrugated boards [18,20] and concrete slabs reinforced with spatial trusses [19]. Furthermore, this numerical technique was modified for beam analysis and was applied for perforated thin-walled cross-sections of Z and C profiles and rectangular pipes [21]. Moreover, it has been shown that the homogenization technique can be used in structural optimization to efficiently obtain solutions. In [9], the geometrical parameters of the thin-walled cross-section were sought by using surrogate models without losing the accuracy of the calculation results. Not only the structural parameters may be optimized but also the material [22,23] or the topology of the structure [24,25].

Typical homogenization techniques are taking into consideration a periodic part of the structure or body. In the calculation, usually the geometry and the material are idealized, i.e., they have no imperfections. Since, for thin-walled structures such as a Z or C profile, even small loading may cause initial instability, so taking imperfections into account when applying the homogenization technique can be crucial for the correct determination of the actual stiffness of the structure, e.g., in the case of the flexural buckling of purlins.

This concern was the motivation to conduct numerical research in this direction in the article. The main purpose of this work is to quantify the reduction in the effective stiffness of a thin-walled beam, taking into account local imperfections due to compression, bending, or shear via the numerical homogenization technique. It is also indicated herein which buckling mode may be representative to be used while taking into account imperfections instead of computing buckling modes for all load-cases separately.

The Materials and Methods chapter consists of four sections. In the first section, the general workflow is described, while in the second section, the mathematical details of the homogenization method used are summarized. In the third section, the buckling analysis from an algorithmic point of view is presented, while in the fourth section, the representative volume element (RVE) models of the Z-profile used in the study are shown. The Results chapter contains the outcomes of the numerical homogenization of the reference Z-profile case and its counterparts for the same beam section with varied imperfections. The comparison to the reference case is also included. In the Discussion and Conclusions chapters, the results of the comparison are commented on, and the final outlines are presented.

This article continues the work on homogenization presented in our previous papers. However, in this case, a thin-walled open section is analyzed, taking into account the geometrical imperfections of the section. In order to correctly introduce imperfections to the model, in the first step, displacements resulting from different buckling modes of the cross-section are introduced into the model, taking into account the selected boundary conditions. Then, the influence of these imperfections on the stiffness drops, which are determined by the numerical homogenization method, is checked. Eventually, a form of imperfection was chosen that was the most representative.

## 2. Materials and Methods

### 2.1. General Information (Workflow)

**Figure 1:**
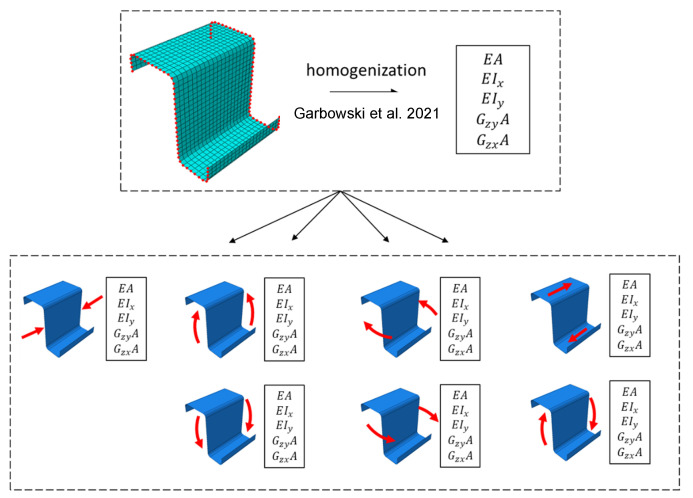
Schematic illustration of the study workflow: shell-to-beam homogenization (Garbowski et al. 2021 [18]) for the reference beam and its counterparts for beams with imperfections due to different modes.

In this study, the influence of the geometric imperfection due to particular deformation mode on the mechanical characteristics of the beam section was computed. The scheme illustrating the study workflow is presented in Figure 1. First, the numerical shell-to-beam homogenization method [18,21] was implemented (see Section 2.2). Thanks to the homogenization, the compressive stiffness (EA), bending stiffness about the horizontal axis ($EI_{x}$), and vertical axis ($EI_{y}$) and shearing in the plane of the web ($G_{zx}A$) and flanges ($G_{zy}A$) were calculated.

Then, by using this method, the selected Z-profile (see Section 2.4) without any imperfection was homogenized, and its representative **ABDR** matrix [18] (according to a lamination theory) was computed—this result is considered in the study as the reference result. Next, the buckling analyses (see Section 2.3) were performed for different deformation modes, received from applying typical loads: compression and bending/shearing forces in two directions. The buckling modes received with different scale ratios were then used to compute the weakened mechanical properties of the beams by applying the homogenization method (see Section 2.4). Later, those results were compared to the reference one in order to select one deformation mode that could be representative for all cases.

### 2.2. Shell-to-Beam Numerical Homogenization

As in [21], in the homogenization process, the basic principle of strain energy equivalence in the idealized and digitized model was also used here. The most widespread and versatile among other methods, the finite element (FE) method was used here for the digitization. The numerical homogenization procedure within this framework is divided into three steps. In the first step, one can identify two phases: first, the FE discretization of the selected RVE is required, and second, the assembly of the full stiffness matrix $\left[K\right]$ of the entire RVE needs to be performed, which is condensed $\left[K_{e}\right]$ later to the active, external nodes only (indicated in the red color in Figure 1). In the notation of the method, e is for external nodes, while i determines the internal nodes.

In the second step, a transformation matrix should be constructed that associates generalized strains and nodal displacements. In the case of shell-to-beam homogenization, this matrix takes the form [21]:

(1) $$u_{i}=H_{i}\varepsilon_{i}.$$

in which u is a displacement vector, and ε is a strain vector, and the $H_{i}$ matrix, adopted for a shell RVE model, can be derived:

(2) $${\left[\begin{array}{c} u_{x}\\ u_{y}\\ u_{z}\\ \theta_{x}\\ \theta_{y} \end{array}\right]}_{i}={\left[\begin{array}{ccccc} 0 & 0 & -\frac{z^{2}}{2} & \frac{z}{2} & 0\\ 0 & -\frac{z^{2}}{2} & 0 & 0 & \frac{z}{2}\\ z & yz & xz & \frac{x}{2} & \frac{y}{2}\\ 0 & 0 & -z & 0 & 0\\ 0 & -z & 0 & 0 & 0 \end{array}\right]}_{i}{\left[\begin{array}{c} \varepsilon_{x}\\ \kappa_{x}\\ \kappa_{y}\\ \gamma_{xz}\\ \gamma_{yz} \end{array}\right]}_{i}.$$

It is worth noting that the above matrix applies to every node (marked in red in Figure 1), and that the *x*, *y*, *z* coordinates refer to the coordinates of all points to which the stiffness matrix of the entire RVE model has been condensed.

In the final step, matrix $\left[H_{i}\right]$ is assemble to the matrix $\left[H_{e}\right]$, and then the effective stiffness can be computed according to the formula [21]:

(3) $$H_{k}=\frac{H_{e}^{T}K_{e}H_{e}}{\left\{length\right\}}=\left[\begin{array}{ccccc} A_{33} & B_{31} & B_{32} & 0 & 0\\ B_{13} & D_{11} & 0 & 0 & 0\\ B_{23} & 0 & D_{22} & 0 & 0\\ 0 & 0 & 0 & R_{44} & 0\\ 0 & 0 & 0 & 0 & R_{55} \end{array}\right]\;.$$

where: $A_{33}$—tensile stiffness along longitudinal axis; $D_{11}$ and $D_{22}$—bending stiffnesses with respect to the in-plane directions; $R_{44}$ and $R_{55}$—shear stiffnesses of RVE and $B_{13}=B_{31}$ and $B_{23}=B_{32}$—the terms of compressive-bending coupling.

If $B_{13}$ and/or $B_{23}$ are not zeros in the $\left[H_{k}\right]$ matrix, it means that the assumed center axis in the homogenized RVE was not aligned with the natural axes. In such case, in order to determine the bending stiffness in the neutral axes, one should use the simple relationship to replace $D_{11}$:

(4) $$D_{11}^{*}=D_{11}-\frac{B_{13}^{2}}{A_{33}},$$

and $D_{22}$:

(5) $$D_{22}^{*}=D_{22}-\frac{B_{23}^{2}}{A_{33}}.$$

### 2.3. Buckling Analysis

The buckling problem solved by the finite element method consists of two steps: pre-buckling static analysis and nominal buckling analysis. In the first step, the global stiffness matrix is computed, $K_{0}$, and then the nodal forces for initial load configuration, $P^{*}$, assuming that $P=\lambda P^{*}$, in which λ is the dimensionless load factor. After taking into account the boundary conditions, the finite element method system of equation is solved, i.e.,

(6) $$K_{0}\;\cdot d^{*}=P^{*}.$$

The nodal displacements are represented by $d^{*}=K_{0}^{-1}\cdot P^{*}$. Based on $d^{*}$, $d^{e*}$ are extracted for each element. Later, the displacement gradients, $g^{e*}$, and generalized stresses, $s^{e*}$, are determined. In the second step, the initial stress matrices for each element are generated, i.e., $K_{\sigma }^{e}\left(s^{e*}\right)$, and then for the overall structure, $K_{\sigma }\left(s^{*}\right)$. Finally, we may mathematically formulate the buckling problem by the expression:

(7) $$[K_{0}+\lambda K_{\sigma }]v=0,$$

in which λ is the eigenvalue, while v is the eigenvector. By solving the problem we determine the eigenvalues $\lambda_{i}$ with counterpart eigenvectors $v_{i}$. In a buckling problem, the eigenvalue obtained is the critical load coefficient (multiplier) and the eigenvector determines the post-buckling deformation mode of the structure, here the RVE of the beam cross-section.

### 2.4. Reference Model and Models with Geometric Imperfections

In this article, the thin-walled cold-formed Z-profile that has been subjected to different types of buckling are analyzed. The influence of geometric imperfections on the local change of the effective stiffness of the Z profile was investigated using the numerical homogenization method, which is described in Section 2.2. In order to use the above method, it is necessary to properly define the stiffness matrix of the representative volumetric element (RVE) of the thin-walled beam considered.

**Figure 2:**
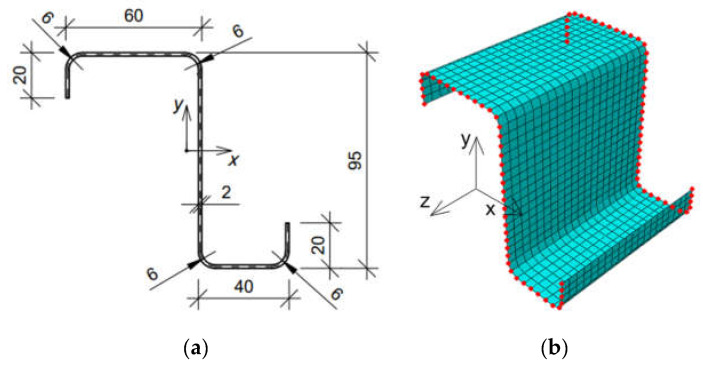
Z profile considered: (**a**) cross-section (units in mm); (**b**) finite element mesh with condensed nodes selected for a 100 mm case.

For this purpose, the RVE of the Z profile with lengths of 100 mm, 150 mm, and 200 mm was built. The cross-section of the beam was modeled as a shell structure with the shape and dimensions shown in Figure 2a. The model was meshed with S4 shell elements, i.e., a 4-node general-purpose shell element (see Abaqus FEA Documentation [26]). The mesh size was 5 mm. Thus, for a model with an elongation of 100 mm, 880 elements were obtained (see Figure 2b); for a length of 150 mm, 1320 elements were obtained, and 1760 elements were obtained for an elongation of 200 mm. The isotropic linear elastic model of steel with the following material parameters was used to describe the material properties: Poisson’s ratio equal to $\nu =0.3\;\left[-\right]$, and Young’s modulus equal to E =210 GPa.

**Figure 3:**
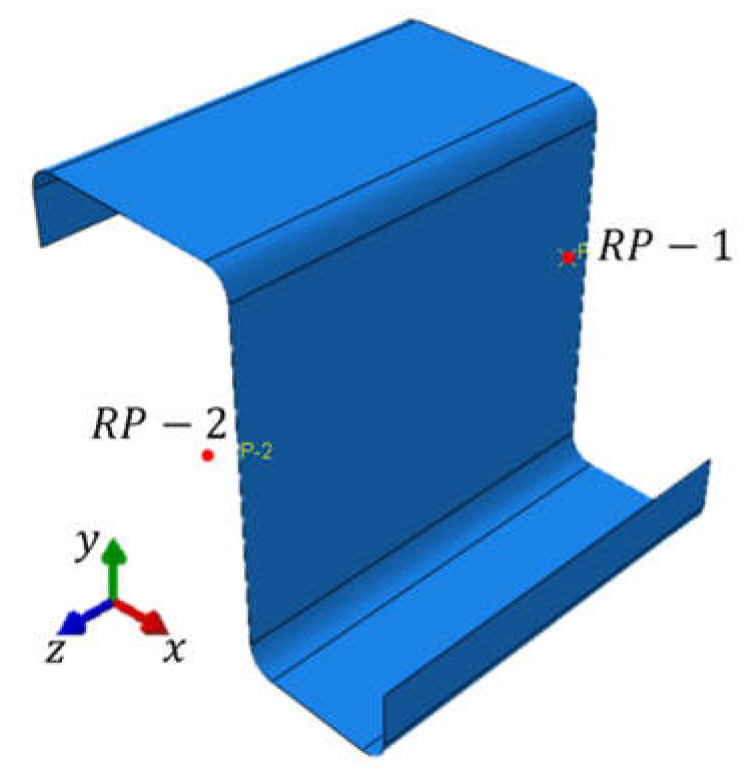
RVE model of the Z profile with the reference points marked in red.

The reference model, i.e., without geometric imperfections, was built as described above and no load nor boundary conditions were applied. On the other hand, in models with imperfections, different deformation modes were enforced to cause the cross-section buckling due to compression, bending about to the horizontal and vertical axis and shearing in the plane of the web and flanges separately. The displacements were applied at the reference points shown in Figure 3 to obtain the particular deformation mode. The reference points were located at the front and rear of the RVE, in the center of gravity of the cross-section. The points were shifted outwards 1 mm along the z axis.

For compression-induced cross-section buckling, three elongation lengths of RVE (100 mm, 150 mm, and 200 mm) were analyzed. The buckling in compression was obtained by applying the displacements along the z axis at the reference points. The enforced displacement at point RP-1 was 0.5 mm, and, at point RP-2, it was −0.5 mm. Additionally, in both points, the displacements in the direction of the x axis, the y axis, and the rotation in relation to the z axis were blocked (assumed to be equal to 0). For the remaining cases, the RVE elongation was assumed to be 100 mm.

While considering buckling in bending about the horizontal axis, two cases were analyzed due to the unequal width of the flanges: (i) the tension of the upper part of the cross-section and (ii) the tension of the lower part of the cross-section. In both cases, the displacements in the reference points along the x axis, y axis, and z axis and rotations in the y axis and z axis were assumed to be 0. In contrast, the rotation about the x-axis for (i) case was 0.5 radians at point RP-1 and −0.5 radians at point RP-2, respectively. For (ii) case of bending about the horizontal axis, the rotation about the x axis for RP-1 was −0.5 radians and for RP-2 was equal to 0.5 radians.

The buckling for bending about the vertical axis was also considered for two load cases: (i) the tension of the right part of the cross-section and (ii) the tension of the left part of the cross-section. For variant (i), the rotation around the y axis in the reference point (RP-1) was assumed to be 0.5 radians and in RP-2 −0.5 radians. For variant (ii), the rotation around the y axis was assumed to be −0.5 radians in RP-1 and 0.5 radians in RP-2. The remaining displacements were blocked at the reference points in two cases.

The next model considered the shear buckling in the plane of the web. For such buckling, two cases were analyzed due to the type of applied load/displacement: (i) the shear of the cross-section in the plane of the web and (ii) the shear in the plane of the flanges. In the model for (i) case, the translational displacements along the x axis and z axis, as well as the rotations around the x and z axes were blocked; the displacements along the y axis were assumed to be 0.5 mm at RP-2 and −0.5 mm for RP-1. However, for (ii) case, the displacements at both reference points were assumed to be equal to 0, except for rotation around x axis, which was equal to −0.5 radians. In the case of shear buckling, in the plane of the flanges, a displacement along the x axis was 0.5 mm at RP-2 and −0.5 mm at RP-1. Displacements along y and z axes and rotations around y and z axes were assumed to be 0.

## 3. Results

This section presents the results obtained from numerical analyses for a thin-walled Z-type profile without holes and with rounded corners. The calculations examined the influence of buckling on the change in the local stiffness characteristics of the beam. Moreover, it was analyzed to what extent the elongation of the profile and the size of imperfections affect the reduction in individual stiffness. The elongation was from 100 mm to 200 mm, and the size of imperfections was from 0 to 5 mm. The size of the imperfections is here the maximal displacement assumed for RVE due to buckling. The analyses were conducted for compression and bending in relation to the horizontal and vertical axis of the cross-section and shear in two planes, vertical and horizontal.

First, the reference stiffness was computed and presented in Table 1 according to the homogenization method described in Section 2.2 [18,21]. The reference results are the one received for the case without buckling included. Table 1 shows the effective stiffness obtained for a Z-profile with a constant mesh size of 5 mm and various elongation lengths. The depth of the sample’s elongation varied from 100 mm to 200 mm. Second, the cases with buckling included were computed. Next, the stiffness reductions in comparison to the reference results were computed and are shown in the tables.

### 3.1. Buckling due to Compression

The changes in stiffness due to compression for the Z-type profile was computed for a fixed mesh with a size of 5 mm and a variable elongation from 100 mm to 200 mm. The influence of imperfection size on the effective stiffness was also investigated.

The individual stiffness drops depending on the elongation and imperfection size for buckling modes 1 and 2 are presented in Table 2 in percentages. The first value in the table for stiffness reduction applies to mode 1, while the second number (after the slash) is the stiffness reduction obtained for mode 2.

**Figure 4:**
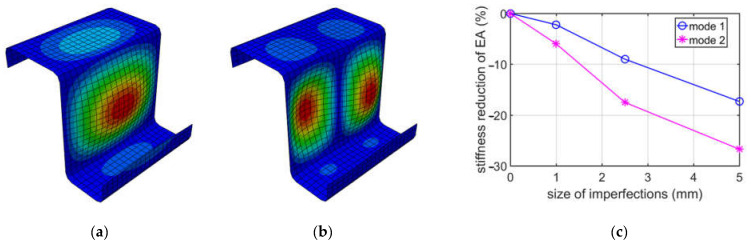
Buckling in compression for 100 mm depth: (**a**) mode 1; (**b**) mode 2; (**c**) plot of the stiffness reduction of EA, depending on the size of imperfections.

For a better illustration of the results due to buckling caused by compression, the decrease in compressive stiffness EA for a Z-profile with an elongation of 100 mm is shown in Figure 4. Additionally, in Figure 4a,b, the buckling of modes 1 and 2 obtained during the compression of the Z-profile are presented.

**Figure 5:**
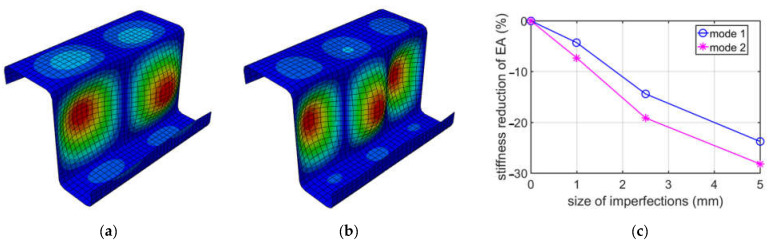
Buckling in compression for 150 mm depth: (**a**) mode 1; (**b**) mode 2; (**c**) plot of the stiffness reduction of EA, depending on the size of imperfections.

On the other hand, the buckling modes 1 and 2 for a Z-profile with an elongation of 150 mm and a decrease in compressive stiffness (EA), depending on the size of the imperfection, are shown in Figure 5.

**Figure 6:**
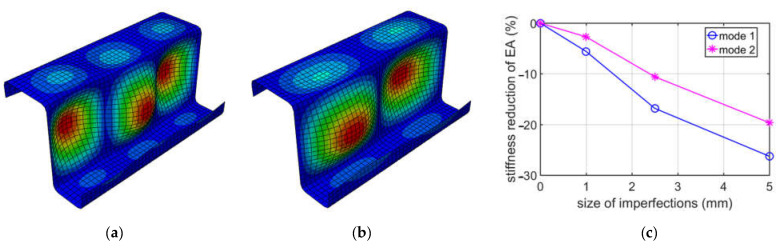
Buckling in compression for 200 mm depth: (**a**) mode 1; (**b**) mode 2; (**c**) plot of the stiffness reduction of EA, depending on the size of imperfections.

In Figure 6, the results due to buckling caused by compression for the Z-type profile with a depth of 200 mm are shown. As previously, the buckling mode 1 and 2 and the compressive stiffness reduction (EA) depending on the size of the imperfection are presented.

### 3.2. Buckling due to Bending about the Horizontal Axis

Buckling due to bending about the horizontal axis of the cross-section was considered for two cases: (i) the tension of the upper part of the cross-section and (ii) the tension of the lower part of the cross-section, because the cross-section considered has no axis of symmetry. For the Z-type cross-section with 100 mm elongation, the influence of the imperfection size on individual stiffness was investigated. The stiffness reduction due to bending for the (i) case is presented in Table 3 in percentages.

**Figure 7:**
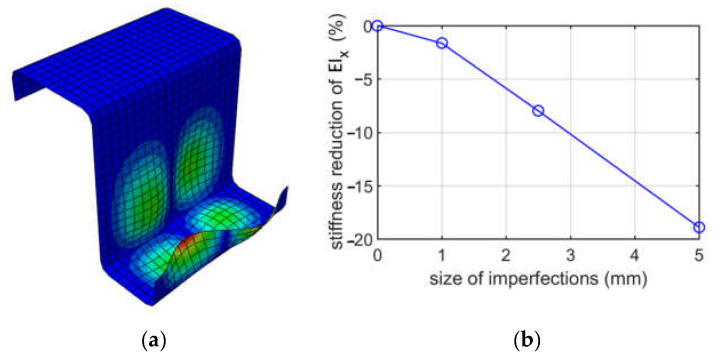
Buckling due to bending about the horizontal axis, top flange in tension ((i) case) for a depth of 100 mm: (**a**) mode 1; (**b**) plot of the stiffness reduction of $EI_{x}$, depending on the size of imperfections.

Figure 7 shows the first buckling mode due to bending for case (i) (tension of the upper part of the cross-section) and the reduction of bending stiffness ${\mathrm{EI}}_{x}$ about the horizontal axis depending on the size of imperfections for a Z-type profile with 100 mm elongation.

Table 4 shows the stiffness drops depending on the size of imperfections for (ii), the case of bending about the horizontal axis, when the lower part of the cross-section is in tension. The results refer to the Z-type profile with 100 mm elongation.

**Figure 8:**
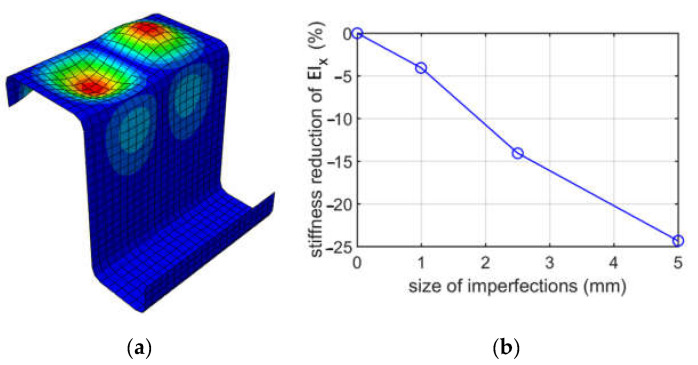
Buckling due to bending about the vertical axis for case (ii) (tension of the lower part of the cross-section), for a depth of 100 mm: (**a**) mode 1; (**b**) plot of the stiffness reduction of $EI_{x}$, depending on the size of imperfections.

Buckling mode 1 for a Z profile with 100 mm elongation due to bending about the horizontal axis and tension of the lower part of the cross-section (case (ii)) is shown in Figure 8. The plot of the reduction in the bending stiffness of ${\mathrm{EI}}_{x}$ depending on the imperfection size is shown in Figure 4.

### 3.3. Buckling due to Bending about the Vertical Axis

Buckling due to bending about the vertical axis was considered for two cases: (i) the tension of the right part of the cross-section and (ii) the tension of the left part of the cross-section. The influence of the size of an imperfection caused by bending on the decrease of individual stiffness for a Z-type cross-section with an elongation of 100 mm was analyzed. Table 5 shows the stiffness reduction due to bending about the vertical axis, i.e., case (i).

**Figure 9:**
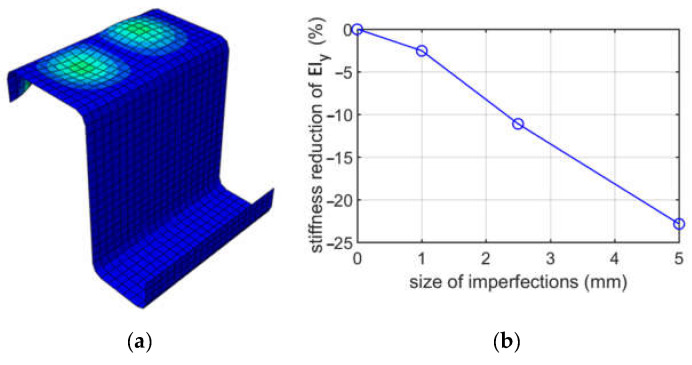
Buckling due to bending about the horizontal axis for (i) case, for a depth of 100 mm: (**a**) mode 1; (**b**) plot of the stiffness reduction of $EI_{y}$, depending on the size of imperfections.

Figure 9 shows the first buckling mode for case (i), i.e., bending about the vertical axis, when the right part of the cross-section is in tension and the plot of the bending stiffness of the $EI_{y}$ reduction depends on the size of imperfections for the Z profile with an elongation of 100 mm.

The stiffness reduction for a Z-profile with 100 mm elongation due to bending about the vertical axis for the case (ii), depending on the value of the imperfection, are presented in Table 6.

**Figure 10:**
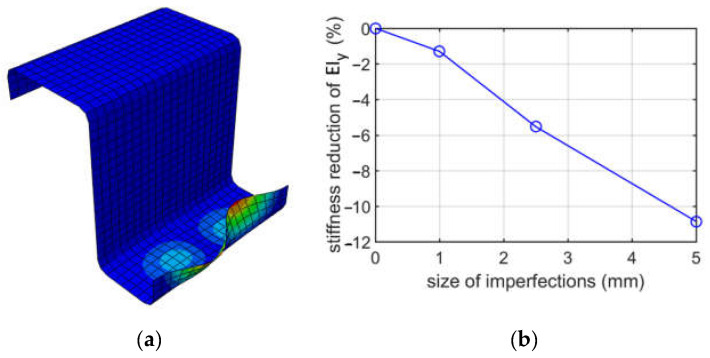
Buckling due to bending about the vertical axis for case (ii), for a depth of 100 mm: (**a**) mode 1; (**b**) plot of the stiffness reduction of $EI_{y}$, depending on the size of imperfections.

In Figure 10, the results due to bending about the vertical axis when the left part of the cross-section is in tension (case (ii)) for a Z-profile with a depth of 100 mm are presented. Figure 10a presents buckling mode 1. The plot of the reduction in the bending stiffness of $EI_{y}$ depending on the size of imperfection is demonstrated in Figure 10b.

### 3.4. Buckling due to Shear

Buckling in shear was analyzed for two variants of load. The shear of the cross-section in the plane of the web was labeled as case (i), while the shear in the plane of the flanges was labeled as case (ii). For these shear variants, the influence of the imperfection size and the method of implementing shear on the change of the stiffness of the Z-profile with 100 mm elongation were investigated.

Table 7 shows the individual stiffness reduction for shearing in the web plane, i.e., case (i). The shearing effect was obtained by two methods. Method I was achieved by applying translational displacements. Method II was obtained by applying rotational displacements. The values from both methods are shown in Table 7, separated by a slash.

**Figure 11:**
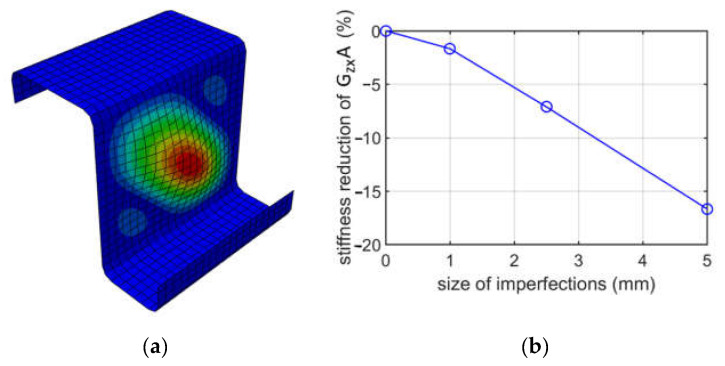
Buckling due to shearing for case (i) for the Z profile with an elongation of 100 mm: (**a**) mode 1; (**b**) plot of the stiffness reduction of $G_{zx}A$, depending on the size of imperfections.

Figure 11 shows the buckling of mode 1 due to shearing in the web plane (case (i) of shearing) and the shear stiffness reduction of $G_{zx}A$ depending on the size of imperfections for the Z-profile with an elongation of 100 mm and method I, i.e., shear caused by displacement.

The decrease in stiffness due to buckling from shearing in the plane of the flanges, i.e., case (ii), the shearing case for the Z profile with an elongation of 100 mm, depending on the imperfection value, is presented in Table 8. As has been previously done, two ways of obtaining shearing deformation were applied, i.e., method I and method II were used. In method I, the shearing is caused by applying translational displacements, and, in method II, the shearing is obtained by applying rotational displacements.

**Figure 12:**
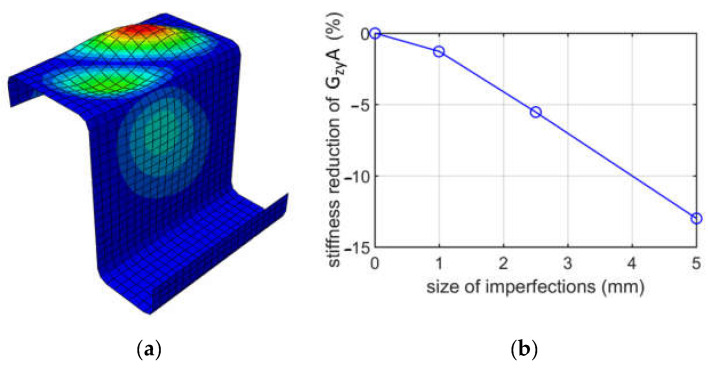
Buckling due to shearing for case (ii), for a depth of 100 mm: (**a**) mode 1; (**b**) plot of the stiffness reduction of $G_{zy}A$, depending on the size of imperfections.

In Figure 12, buckling mode 1 and a plot of the stiffness reduction of $G_{zy}A$ caused by shearing in the plane of the flanges (case (ii)) are presented for the Z profile with elongation of 100 mm and method I, i.e., shear caused by applying translational displacements.

## 4. Discussion

The systematic numerical studies of homogenization adopted on an cold-formed unsymmetric beam profile to obtain its representative stiffnesses allows a comparison of the results between different buckling modes used, as well as the size of imperfections applied, or the method used to determine a particular deformation.

While analyzing buckling due to compression, see Table 2, it appeared that the first two deformation modes may be important, because their eigenvalues were similar, i.e., the difference between them was no more than 10%. The first mode has a more global shape, with one extreme at the web, while the second mode is more local, that is, it has two extremes at the web. The direct values of stiffness reductions are different when comparing mode 1 and mode 2; see Table 2. However, it should be noted that the factor of imperfection (Δd/L) in mode 1 and mode 2 are different. Δd is the relative displacement, and L is the elongation depth. For instance, in mode 1, the factor is equal to 1/100 for 1 mm, and, for mode 2, it is equal to 2/100 also for 1 mm. Thus, the comparable results in our summary (Figure 2) for the EA is mode 1 for a 2.5 mm imperfection and mode 2 for a 5.0 mm imperfection. Those two values are equal to 17.52% and 17.34%, respectively, and are close. A similar effect may be observed for other elongation depths. It is worthwhile to note that, for 150 mm, the deformations for mode 1 and mode 2 are flipped. They are very much similar, however, in the 150 mm case; mode 1 has two extremes, and mode 2 has three extremes, while, in the 200 mm case, mode 1 has three extremes, and mode 2 has two extremes. If one would analyze the influence of the size of imperfections on EA stiffness reduction from an engineering point of view, it is approximately linear, if the imperfection factor is taken into consideration, as is presented in Figure 3, Figure 4 and Figure 5.

When analyzing buckling due to bending about the horizontal axis, two cases were considered, that is, the top flange in tension and the lower flange in tension. The upper flange has a 60 mm width, while the lower one has a 40 mm width. Such a width difference resulted in obtaining different modes; in case (i), the extremes were achieved in the lower flange, and in case (ii), the extremes were obtained in the upper flange. The stiffness reductions of $EI_{x}$ were also different, and much larger reductions were achieved for case (ii). For case (i), the stiffness reductions were from 1.66% to 18.9%, depending on the size of imperfections assumed (1.0, 2.5, or 5.0 mm), while for case (ii), the reductions were from 4.08% up to 24.34%. Stiffness reductions in case (ii) were from 1.3 up to 2.5 times larger than in case (i). This effect could be expected because, in case (ii), the deformation extremes are in the compressed flange, while in case (i), they occur in a less important flange stiffener.

When analyzing buckling due to bending about the vertical axis, two cases were considered, that is, the left stiffener in compression and the right stiffener in compression. Both stiffeners have the same height, i.e., 20 mm. Difference in flanges width resulted in obtaining different modes; see Figure 8 and Figure 9; in both cases, the extremes were similarly located on the compressed stiffeners. The stiffness reductions of $EI_{y}$ were also different; see Table 5 and Table 6; much larger reductions were achieved for case (i). For case (i), the stiffness reductions were from 2.53% to 22.83%, depending on the size of imperfections assumed (1.0, 2.5 or 5.0 mm), while for case (ii), the reductions were from 1.3% up to 10.86%. The stiffness reductions for case (i) were approximately twice as large as for case (ii). This effect could be expected because, in case (i), the stiffener supports the larger width flange and because its deterioration due to imperfection decreases the $EI_{y}$ stiffness more than in case (ii).

When analyzing buckling due to shearing, two cases were considered, that is, shearing in the zx plane and shearing in the zy plane, see Figure 1. Moreover, for each case, two methods of applying shear were considered. For shearing in the zx plane (shearing web), the results of two methods give values with a negligible difference; see Figure 10. For instance, for $G_{zx}A$ and a 1.0 mm imperfection, the reduction percentages are 1.60% and 1.68%, which gives a 5% difference. The diagonal deformation of the web was achieved. For shearing in the zy plane (shearing flanges), the results of two methods also give values with a negligible difference; see Figure 11. For instance, for $G_{zy}A$ and a 2.5 mm imperfection, the values are 5.25% and 5.53%, which gives a 5.3% difference; see Table 7 and Table 8. The diagonal deformation of the flange was also achieved. The conclusion from shearing analyses is that both methods for zx and zy plane shearing, are equally good. Furthermore, as previously, from an engineering point of view, the influence of the size of imperfections on the $G_{zy}A$ or $G_{zy}A$ stiffness reduction is approximately linear.

In this paper, we presented a methodology on how to compute the deteriorated characteristics of a beam section due to several types of imperfections. The methodology requires solving several buckling problems, with different loads and numerical homogenization [18,21] for each case. Despite a relatively small time cost, it requires a lot of modeling work, i.e., defining various boundary conditions, building separate models, etc. According to the results of our calculations presented in Section 3, this effort can be limited to modeling only the case of bending about the horizontal axis and shearing of flanges. Bending about the horizontal axis serves for obtaining all stiffness reductions apart from the shearing of the flanges. Therefore, the reduction of $E_{x}I$ would be exact from the horizontal bending case. The reduction of $G_{zy}A$ would be exact from the shearing of the flanges. The rest of the stiffness reductions would be taken as the approximated values from the horizontal bending case. Such an approach would reduce the effort needed to determine the characteristics of a beam section due to various types of imperfections.

The conclusions presented applies to the cross-sections analyzed. To adopt those findings to another types of cross-section, verification simulations are recommended to be performed. Another limitation is that the buckling results depend on the ratio between the dimensions of flanges/webs and its extrusion, while considering a RVE approach.

## 5. Conclusions

In the paper, a methodology for numerically determining the deteriorated properties of a beam section due to imperfections was presented. Thin-walled Z-type beams with variable elongation and a different load pattern were analyzed. The analyses conducted use a method of numerical shell-to-beam homogenization by using the principle of the elastic equilibrium of the strain energy.

In particular, this paper shows what kind of local imperfections deteriorate the effective stiffness of the cross-section, which so far has not been taken into account. The results presented in the study directly help to build engineering intuition without conducting complex finite element computations. Moreover, the algorithm proposed may serve to compute the effective stiffnesses for other cross-sections, such as a C or Sigma profile. In this research paper, first, the homogenization method was used to calculate the mechanical parameters of an undeformed beam (reference case). Next, in the second part, the buckling analyses for the RVE model were subjected to various types of loading (compression, bending in reference to two axes, and shear in two planes). Finally, the obtained buckling modes were used to calculate the individual stiffness drops. In the end, an alternative, faster, and simplified approach was proposed that gave satisfactory results compared to the full methodology.

## Figures and Tables

**Table 1:** Effective stiffness of the Z profile with a 5 mm mesh depending on the elongation (beam axis).

Depth $\left(mm\right)$	EA $\left(10^{7}\;Pa\;m^{2}\right)\;$	$EI_{y}\;$ $\left(10^{4}\;Pa\;m^{4}\right)\;$	$EI_{x}\;$ $\left(10^{5}\;Pa\;m^{4}\right)\;$	$G_{zy}A\;$ $\left(10^{6}\;Pa\;m^{2}\right)\;$	$G_{zx}A\;$ $\left(10^{7}\;Pa\;m^{2}\right)\;$
200	9.135	6.571	1.271	6.037	9.350
150	9.160	6.542	1.269	7.604	1.098
100	9.211	6.521	1.270	9.801	1.308

**Table 2:** Stiffness reduction of the Z profile depending on the elongation depth (beam axis) and buckling mode in compression.

Depth $\left(mm\right)$	Size of Imperfections $\left(mm\right)$	Stiffness Reduction (Mode 1/Mode 2)				
		EA $\left(\%\right)\;$	$EI_{y}\;$ $\left(\%\right)\;$	$EI_{x}\;$ $\left(\%\right)\;$	$G_{zy}A\;$ $\left(\%\right)\;$	$G_{zx}A\;$ $\left(\%\right)\;$
100	1.0	−2.22/−5.98	0.04/−0.36	−0.19/−0.71	−0.05/−0.14	−0.32/−2.44
	2.5	−9.00/−17.51	−0.27/−1.32	−1.14/−3.30	−0.31/−0.82	−1.81/−12.13
	5.0	−17.34/−26.73	−1.15/−2.81	−3.86/−7.97	−1.09/−2.78	−6.38/−30.39
150	1.0	−4.31/−7.23	−0.27/−0.31	−0.40/−0.94	−0.23/−0.39	−1.49/−2.72
	2.5	−14.40/−19.14	−1.17/−1.35	−2.18/−3.90	−1.33/−1.96	−8.25/−13.46
	5.0	−23.79/−28.22	−2.92/−3.13	−6.38/−9.10	−4.46/−5.89	−24.15/−33.63
200	1.0	−5.59/−2.71	−0.25/−0.17	−0.64/−0.22	−0.50/−0.25	−1.90/−0.90
	2.5	−16.83/−10.59	−1.26/−0.82	−2.95/−1.28	−2.47/−1.46	−10.13/−5.30
	5.0	−26.28/−19.66	−3.24/−2.25	−7.84/−4.28	−7.44/−5.04	−28.05/−17.52

**Table 3:** Stiffness reduction of a Z profile with an elongation of 100 mm due to bending about the horizontal axis for (i) case, depending on the size of imperfections.

Size of Imperfections $\left(mm\right)$	Stiffness Reduction				
	EA $\left(\%\right)\;$	$EI_{y}\;$ $\left(\%\right)\;$	$EI_{x}\;$ $\left(\%\right)\;$	$G_{zy}A\;$ $\left(\%\right)\;$	$G_{zx}A\;$ $\left(\%\right)\;$
1.0.	−2.47	−1.79	−1.66	−0.21	−0.79
2.5	−10.85	−7.91	−7.99	−1.19	−4.57
5.0	−21.95	−16.40	−18.90	−3.67	−14.59

**Table 4:** Stiffness reduction of Z profile with an elongation of 100 mm due to bending about the horizontal axis for case (ii), i.e., the tension of the lower part of the cross-section, depending on the size of imperfections.

Size of Imperfections $\left(mm\right)$	Stiffness Reduction				
	EA $\left(\%\right)\;$	$EI_{y}\;$ $\left(\%\right)\;$	$EI_{x}\;$ $\left(\%\right)\;$	$G_{zy}A\;$ $\left(\%\right)\;$	$G_{zx}A\;$ $\left(\%\right)\;$
1.0	−3.51	−3.64	−4.08	−1.22	−0.30
2.5	−11.76	−13.89	−14.06	−6.57	−1.54
5.0	−20.67	−26.44	−24.34	−18.37	−4.73

**Table 5:** Stiffness reduction of Z profile with an elongation of 100 mm due to bending about the vertical axis for (i) case, depending on the size of imperfections.

Size of Imperfections $\left(mm\right)$	Stiffness Reduction				
	EA $\left(\%\right)\;$	$EI_{y}\;$ $\left(\%\right)\;$	$EI_{x}\;$ $\left(\%\right)\;$	$G_{zy}A\;$ $\left(\%\right)\;$	$G_{zx}A\;$ $\left(\%\right)\;$
1.0	−1.04	−2.53	−0.95	−0.28	−0.08
2.5	−4.70	−11.11	−4.64	−1.63	−0.45
5.0	−10.22	−22.83	−11.48	−5.43	−1.36

**Table 6:** Stiffness reduction of the Z profile with elongation of 100 mm due to bending about the vertical axis for (ii) case, depending on the size of imperfections.

Size of Imperfections $\left(mm\right)$	Stiffness Reduction				
	EA $\left(\%\right)\;$	$EI_{y}\;$ $\left(\%\right)\;$	$EI_{x}\;$ $\left(\%\right)\;$	$G_{zy}A\;$ $\left(\%\right)\;$	$G_{zx}A\;$ $\left(\%\right)\;$
1.0	−0.54	−1.30	−0.57	−0.10	−0.06
2.5	−2.32	−5.53	−2.56	−0.56	−0.34
5.0	−4.83	−10.86	−5.75	−1.71	−1.01

**Table 7:** Stiffness reduction of the Z profile with elongation of 100 mm due to shearing for case (i), depending on the size of imperfections.

Size of Imperfections $\left(mm\right)$	Stiffness Reduction (Method I/Method II)				
	EA $\left(\%\right)\;$	$EI_{y}\;$ $\left(\%\right)\;$	$EI_{x}\;$ $\left(\%\right)\;$	$G_{zy}A\;$ $\left(\%\right)\;$	$G_{zx}A\;$ $\left(\%\right)\;$
1.0	−2.30/−2.39	−0.02/−0.04	−0.25/−0.26	−0.09/−0.09	−1.60/−1.68
2.5	−8.34/−8.53	−0.25/−0.28	−1.28/−1.34	−0.43/−0.43	−6.65/−7.12
5.0	−14.87/−15.03	−0.56/−0.60	−3.44/−3.47	−1.26/−1.24	−15.18/−16.68

**Table 8:** Stiffness reduction of the Z profile with an elongation of 100 mm due to shearing for case (ii), depending on the size of imperfections.

Size of Imperfections $\left(mm\right)$	Stiffness Reduction (Method I/Method II)				
	EA $\left(\%\right)\;$	$EI_{y}\;$ $\left(\%\right)\;$	$EI_{x}\;$ $\left(\%\right)\;$	$G_{zy}A\;$ $\left(\%\right)\;$	$G_{zx}A\;$ $\left(\%\right)\;$
1.0	−1.66/−1.71	−2.05/−1.91	−1.74/−1.70	−1.18/−1.28	−0.11/−0.14
2.5	−7.11/−6.89	−8.55/−7.49	−7.46/−7.04	−5.25/−5.53	−0.82/−0.91
5.0	−15.00/−14.47	−18.31/−16.21	−15.16/−14.03	−12.48/−12.99	−2.77/−3.00

## Data Availability

The data presented in this study are available on request from the corresponding author.
